# Non-alcoholic fatty liver is associated with increased risk of irritable bowel syndrome: a prospective cohort study

**DOI:** 10.1186/s12916-022-02460-8

**Published:** 2022-08-22

**Authors:** Shanshan Wu, Changzheng Yuan, Zhirong Yang, Si Liu, Qian Zhang, Shutian Zhang, Shengtao Zhu

**Affiliations:** 1 Department of Gastroenterology, National Clinical Research Center for Digestive Disease, Beijing Digestive Disease Center, Beijing Key Laboratory for Precancerous Lesion of Digestive Disease, Beijing Friendship Hospital, Capital Medical University, Beijing, 100050 China; 2 School of Public Health, Zhejiang University School of Medicine, Hangzhou, Zhejiang, 310058 China; 3 Department of Nutrition, Harvard T.H. Chan School of Public Health, Boston, MA 02115 USA; 4 Shenzhen Institute of Advanced Technology, Chinese Academy of Sciences, Shenzhen, 518055 China; 5 Primary Care Unit, Department of Public Health and Primary Care, School of Clinical Medicine, University of Cambridge, Cambridge, CB18RN UK

**Keywords:** Non-alcoholic fatty liver disease, Irritable bowel syndrome, Cohort study

## Abstract

**Background:**

The relationship between non-alcoholic fatty liver degree as well as non-alcoholic fatty liver disease (NAFLD) and irritable bowel syndrome (IBS) remains poorly understood. We aimed to investigate the prospective association of non-alcoholic fatty liver degree as well as NAFLD with incident IBS in a large-scale population-based cohort.

**Methods:**

Participants free of IBS, coeliac disease, inflammatory bowel disease, alcoholic liver disease, and any cancer at baseline from the UK Biobank were included. Non-alcoholic fatty liver degree was measured by a well-validated fatty liver index (FLI), with FLI ≥ 60 as an indicator of NAFLD. Primary outcome was incident IBS. Cox proportional hazard model was used to investigate the associated risk of incident IBS.

**Results:**

Among 396,838 participants (mean FLI was 48.29 ± 30.07), 153,203(38.6%) were with NAFLD diagnosis at baseline. During a median of 12.4-year follow-up, 7129 cases of incident IBS were identified. Compared with non-NAFLD, NAFLD patients showed a 13% higher risk of developing IBS (HR = 1.13, 95%CI: 1.05–1.17) after multivariable adjustment. Compared with the lowest, the highest FLI quartile was associated with a significantly increased risk of IBS (HR_Q4 VS Q1_ = 1.21, 1.13–1.30, *P*_trend_ < 0.001). Specifically, the positive association between non-alcoholic fatty liver degree and IBS was also observed by per SD change of FLI (adjusted HR = 1.08, 1.05–1.10). Further sensitivity analysis and subgroup analysis indicated similar results, with the positive association particularly observed in females, but not in males.

**Conclusions:**

High degree of non-alcoholic fatty liver as well as non-alcoholic fatty liver disease is associated with increased risk of incident IBS. Further studies are warranted to confirm the findings and elucidate the underlying biological mechanisms.

**Supplementary Information:**

The online version contains supplementary material available at 10.1186/s12916-022-02460-8.

## Background

Irritable bowel syndrome (IBS) is a common disorder of gut-brain interaction, characterized by recurrent abdominal pain accompanied by altered bowel habits and bloating without any organic lesions [1, 2]. Recent global epidemiological study reported an estimated 10.1% and 4.1% of the population suffered from IBS according to Rome III and IV criteria, respectively [3]. IBS remains a major disorder associated with reduced health-quality of life, leading to considerable medical costs and a significant economic burden to both patients and the whole society [4, 5]. Therefore, it is of high priority to identify contributing factors, particularly modifiable lifestyle risk factors, to help develop targeted prevention strategies. However, to date, such evidence is limited.

Non-alcoholic fatty liver disease (NAFLD) is defined as excessive hepatic steatosis in the absence of specific causes (i.e., alcohol consumption, hepatitis B or C infection) [6]. Approximately 25% of adult population is affected by NAFLD currently, with worsening epidemic in recent decades coinciding with rising IBS incidence [3, 7, 9]. The factors for pathogenesis of NAFLD are not well clarified. Previous findings suggest insulin resistance, metabolic syndrome, obesity as well as inflammation are involved in the process [8–10]. Growing evidence by several animal and vitro experimental studies supports a plausible link between NAFLD and IBS, including shared proinflammatory cytokines (i.e., increased expressions of tumor necrosis factor (TNF)-α, interleukin (IL)-6, IL-8 and IL-1β, decreased levels of IL-10), cross-talk of liver-brain-gut neural arc and gut-liver axis, dysfunction of intestinal microbiota and impaired intestinal barrier as well as intestinal dysmotility [11–17]. Investigation of fatty liver degree and NAFLD associated with IBS risk is an urgent need considering growing disease burden of IBS. However, to the best of our knowledge, there are no prior epidemiological studies examining NAFLD as well as the degree of fatty liver in relation to risk of incident IBS. Whether fatty liver could affect functions of the gastrointestinal tract and further lead to syndromic manifestations typical of IBS remains to be answered yet.

To address these knowledge gaps, we prospectively investigated the association of non-alcoholic fatty liver degree, as well as NAFLD, with risk of incident IBS in a large population-based long-term follow-up UK cohort.

## Methods

### Study population

This study population was composed of over 500,000 participants from an ongoing large-scale prospective cohort, UK Biobank (UKB). Briefly, participants ranging from 37 to 73 years of age from 22 assessment centers across England, Wales, and Scotland were enrolled between 2006 and 2010. All participants completed baseline questionnaires with anthropometric assessments and reported medical conditions. Details of UKB design were described elsewhere [18]. The study was approved by North West Multicenter Research Ethical Committee, and all participants signed written informed content.

**Fig. 1:**
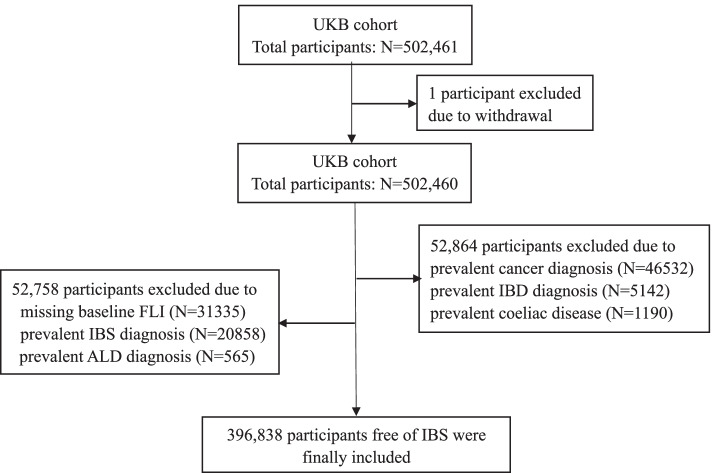
Flowchart of the study population. UKB: UK Biobank; IBS: irritable bowel syndrome; IBD: inflammatory bowel disease; ALD: alcoholic liver disease; FLI: fatty liver index

Participants who were free of IBS with an available non-alcoholic fatty liver index at enrollment were included in this study. Those who already had cancer, inflammatory bowel disease (IBD), alcoholic liver disease (ALD), or coeliac disease diagnosis at enrollment were excluded. All diagnoses were identified through International Classification of Disease-10 (ICD-10) codes (Additional file 1: Table S1). Additionally, 1 participant withdrawal from UKB was excluded. Therefore, a total of 396,838 participants were included in the final analysis. Flowchart of participant selection was listed in Fig. 1.

### Assessment of baseline non-alcoholic fatty liver degree and NAFLD

As no imaging, ultrasonography, or histological data regarding fatty liver was available in the large-scale UKB cohort, we used a well-established index, fatty liver index (FLI), to measure the degree of non-alcoholic fatty liver [19]. Briefly, FLI was calculated through four variables including BMI, waist circumstance (WC), triglycerides (TG), and gamma-glutamyltransferase (GGT) using a previously published and validated regression model [19]. It has been proved to be a reliable index with good discrimination performance of liver ultrasonography-determined NAFLD [area under the receiver operator curve, AUROC = 0.85 (95%CI: 0.81–0.88)] and transient elastography-determined NAFLD (AUROC = 0.85), which has been externally validated and widely accepted in a population-based study [19–21]. Meanwhile, the weighted percent-agreement between FLI and transient elastography was as high as 75.11% (95%CI: 75.10%-75.12%) when validated in a nationally representative sample of the western general population rather than a clinical population [21]. We classified FLI according to quartile distribution with the lowest quartile group as the reference group and the other three quartile groups as exposure groups. Moreover, we also used NAFLD diagnosis or not according to a predefined cutoff, with FLI ≥ 60 as an indicator of NAFLD [19]. Participants who had baseline FLI < 60 were considered in the non-exposure group (non-NAFLD group), while others who were diagnosed as NAFLD were considered in the exposure group (NAFLD group). Further, NAFLD patients with BMI < 25 kg/m^2^ and ≥ 25 kg/m^2^ would be defined as lean and non-lean NAFLD, respectively. Accordingly, NAFLD patients with BMI ≥ 30 kg/m^2^ and < 30 kg/m^2^ would be considered as obese and non-obese NAFLD, separately [22, 23]. Besides, in order to examine the impact of fatty liver measurement on our findings, another well-established index, hepatic steatosis index (HSI), was used to define NAFLD in sensitivity analyses. HSI could be calculated as 8* (serum alanine aminotransferase (ALT)/aspartate aminotransferase (AST) ratio) + BMI (+ 2, if female; + 2, if type 2 diabetes) [24]. An HSI > 36 was defined as an indicator of NAFLD [24].

### Ascertainment of outcome

Primary endpoint was incident IBS, which was determined via ICD-10 codes (K58, Additional file 1: Table S1). IBS diagnosis was based on self-report or linkage to primary care and/or hospital admission data with a censoring date of June 2021.

### Covariates

Based on epidemiological evidence, some sociodemographic characteristics, health behaviors, and comorbidities at baseline were adjusted as covariates [1, 4, 16, 17]. Potential confounders included age (continuous variable), gender (male or female), ethnicity (white or nonwhite), socioeconomic status, education level, smoking status (never, current, or previous), alcohol drinking (never, current, or previous), type 2 diabetes (Yes or No) and physical activity. Socioeconomic status was based on the Townsend deprivation index, which was calculated immediately prior to participants joining UKB using preceding national census output areas [25]. Townsend deprivation index for socioeconomic deprivation was divided into four quartiles. Education was based on self-report of the highest qualification achieved and classified as university or non-university. Physical activity was self-reported and divided into three levels (high, moderate, and low) based on IPAQ (International Physical Activity Questionnaire).

### Statistical analysis

Incidence rate with 95% confidence interval (CI) of IBS was calculated as a number of events per 1000 person-years through Poisson regression. The 12-year cumulative incidence of IBS was calculated by the Kaplan–Meier method. Cox proportional hazard model was conducted to examine the association between fatty liver and incident IBS. The follow-up period started from baseline to the date of first IBS diagnosis or censored at end of the study (June 2021), date of death, or lost-to-follow-up for participants who did not develop IBS. Considering a very small percentage of missing values (0.1–1.2% for all covariates were missing), missing indicators were used.

For FLI quartiles, per standard deviation (SD) change of FLI and diagnosis of NAFLD or not according to predefined cutoff, three multivariable models in addition to univariable analysis were accomplished: model 1, adjusted for age and gender; model 2, additionally adjusted for Townsend deprivation index, education level, ethnicity, smoking status, and alcohol drinking; model 3, additionally adjusted for physical activity and type 2 diabetes. Moreover, restricted cubic spline analysis was conducted to examine the potential non-linear association between baseline FLI and incident IBS, with knots placed at 10th, 50th, and 90th percentiles and the median value of baseline FLI (46.55) as a reference point. Furthermore, subgroup analysis was performed to investigate whether the association between the degree of non-alcoholic fatty liver as well as NAFLD and IBS varied by age (< 45 years, 45-64 years, ≥ 65 years), gender, alcohol drinking, and smoking status. Effect modification was also detected by adding interaction terms of each stratified variable (i.e., age, gender, alcohol drinking, smoking status) and non-alcoholic fatty liver exposure (i.e., FLI quartiles, per SD change of FLI, diagnosis of NAFLD or not). Further analyses were conducted to investigate the association between NAFLD type (lean/non-lean, non-obese/obese NAFLD) and risk of IBS.

In order to assess the robustness of the results, several sensitivity analyses were conducted. Firstly, we excluded participants who had an IBS diagnosis within 1 or 2 years after recruitment respectively, in order to avoid detection bias. Secondly, to rule out the influence of alcohol intake on the non-alcoholic fatty liver during the whole follow-up period, incident ALD cases after baseline were further excluded. Thirdly, the competing risk model by considering lost-to-follow-up and death as competing events were conducted, since those participants might develop IBS thereafter. Fourthly, participants who had hepatitis B/C virus seropositivity were excluded. Fifthly, we additionally adjusted psychologic disorder including depression and anxiety as confounders. Finally, an age-matched (1:1 matching, ± 2 years) cohort was generated as the new dataset to further investigate the association between NAFLD and IBS.

Additionally, sensitivity analyses were conducted by using HSI [diagnosis of NAFLD or not according to predefined cutoff (HSI > 36), per SD change] via adjusted model 3, with additional similar analyses by excluding incident IBS cases within 1 or 2 years after baseline, excluding incident ALD cases, excluding participants with hepatitis B/C virus seropositivity or performing competing risk model.

A two-tailed *P* value < 0.05 was considered to be statistically significant. All analyses were conducted using SAS software Version 9.4 and R version 4.0.2 (forestplot, tableone, ggplot2, and survival packages).

## Results

### Baseline characteristics

Of all 396,838 participants, 47.8% were male. The mean (SD) age was 56.22 (8.11) years at enrollment. The mean FLI was 48.29 ± 30.07. Overall, 153,203(38.6%) participants had a NAFLD diagnosis (FLI ≥ 60) before or at enrollment. Participants in the highest quartile of FLI were more likely to be male, have a lower education level, a lower level of socioeconomic deprivation, higher BMI and WC, a higher level of TG, GGT, and ALT and a higher proportion of prevalent diabetes (Table 1). Baseline characteristics of the study cohort according to the diagnosis of NAFLD or not were listed in Additional file 1: Table S2. Median follow-up period was 12.4 years (interquartile range: 11.6–13.1 years).

**Table 1:** Baseline characteristics according to baseline fatty liver index in the UK Biobank cohort

Characteristic	Total (*N* = 396,838)	Quartile 1 (*N* = 98,371)	Quartile 2 (*N* = 99,289)	Quartile 3 (*N* = 99,714)	Quartile 4 (*N* = 99,464)	*P* value
Age (years)^a^	56.22 ± 8.11	54.22 ± 8.21	56.63 ± 8.10	57.19 ± 7.98	56.83 ± 7.82	< 0.001
Gender						< 0.001
Male	189,759 (47.8)	18,005 (18.3)	44,415 (44.7)	60,465 (60.6)	66,874 (67.2)	
Female	207,079 (52.2)	80,366 (81.7)	54,874 (55.3)	39,249 (39.4)	32,590 (32.8)	
Ethnicity						< 0.001
Non-White	22,788 (5.7)	4840 (4.9)	6078 (6.1)	6445 (6.5)	5425 (5.5)	
White	372,582 (93.9)	93,243 (94.8)	92,834 (93.5)	92,871 (93.1)	93,634 (94.1)	
Unknown	1468 (0.4)	288 (0.3)	377 (0.4)	398 (0.4)	405 (0.4)	
Education level						< 0.001
Non-university	261,356 (65.9)	56,972 (57.9)	63,886 (64.3)	67,902 (68.1)	72,596 (73.0)	
University	130,800 (33.0)	40,552 (41.2)	34,307 (34.6)	30,466 (30.6)	25,475 (25.6)	
Unknown	4682 (1.2)	847 (0.9)	1096 (1.1)	1346 (1.3)	1393 (1.4)	
Townsend deprivation index						
Mean (SD)	− 1.30 (3.09)	− 1.51 (2.97)	− 1.46 (3.02)	− 1.35 (3.08)	− 0.90 (3.24)	< 0.001
Q1(≤ − 3.63)	99,950 (25.2)	26,497 (26.9)	26,241 (26.4)	25,483 (25.6)	21,729 (21.8)	< 0.001
Q2(− 3.63 to − 2.12)	99,303 (25.0)	25,220 (25.6)	25,605 (25.8)	25,311 (25.4)	23,167 (23.3)	
Q3(− 2.12–0.58)	99,259 (25.0)	24,667 (25.1)	24,710 (24.9)	24,739 (24.8)	25,143 (25.3)	
Q4 (> 0.58)	97,832 (24.7)	21,869 (22.2)	22,617 (22.8)	24,047 (24.1)	29,299 (29.5)	
Unknown	494 (0.1)	118 (0.1)	116 (0.1)	134 (0.1)	126 (0.1)	
Smoking status						< 0.001
Never	218,022 (54.9)	61,575 (62.6)	57,310 (57.7)	52,689 (52.8)	46,448 (46.7)	
Previous	135,124 (34.1)	27,564 (28.0)	31,237 (31.5)	35,645 (35.7)	40,678 (40.9)	
Current	41,725 (10.5)	8894 (9.0)	10,288 (10.4)	10,857 (10.9)	11,686 (11.7)	
Unknown	1967 (0.5)	338 (0.3)	454 (0.5)	523 (0.5)	652 (0.7)	
Alcohol drinking						< 0.001
Never	17,303 (4.4)	4221 (4.3)	4224 (4.3)	4365 (4.4)	4493 (4.5)	
Previous	13,388 (3.4)	3058 (3.1)	2957 (3.0)	3270 (3.3)	4103 (4.1)	
Current	365,177 (92.0)	90,914 (92.4)	91,874 (92.5)	91,812 (92.1)	90,577 (91.1)	
Unknown	970 (0.2)	178 (0.2)	234 (0.2)	267 (0.3)	291 (0.3)	
IPAQ						< 0.001
Low	59,637 (15.0)	10,788 (11.0)	12,741 (12.8)	15,232 (15.3)	20,876 (21.0)	
Moderate	131,111 (33.0)	33,043 (33.6)	32,875 (33.1)	33,318 (33.4)	31,875 (32.0)	
High	131,356 (33.1)	37,193 (37.8)	35,528 (35.8)	32,338 (32.4)	26,297 (26.4)	
Unknown	74,734 (18.8)	17,347 (17.6)	18,145 (18.3)	18,826 (18.9)	20,416 (20.5)	
BMI						< 0.001
< 18.5 kg/m^2^	1896 (0.5)	1858 (1.9)	31 (0.0)	7 (0.0)	0 (0.0)	
18.5–24.9 kg/m^2^	124,057 (31.3)	79,531 (80.8)	35,633 (35.9)	8257 (8.3)	636 (0.6)	
25.0–29.9 kg/m^2^	171,674 (43.3)	16,904 (17.2)	59,704 (60.1)	68,734 (68.9)	26,332 (26.5)	
≥ 30 kg/m^2^	99,211 (25.0)	78 (0.1)	3921 (3.9)	22,716 (22.8)	72,496 (72.9)	
Diabetes	10,014 (2.5)	437 (0.4)	1159 (1.2)	2281 (2.3)	6137 (6.2)	< 0.001
WC (cm)^a^	90.47 (13.43)	75.31 (6.29)	86.02 (6.01)	94.10 (6.20)	106.24 (9.84)	< 0.001
TG (mg/dL)^a^	154.40(91.02)	90.82 (33.99)	127.80(51.69)	168.45(73.41)	229.75(115.16)	< 0.001
GGT (U/L)^b^	26.40 (18.60, 41.00)	17.30 (14.10, 22.20)	23.00 (18.00, 31.10)	30.60 (23.00, 43.50)	44.30 (30.80, 69.10)	< 0.001
ALT (U/L)^b^	20.25 (15.47, 27.57)	15.50 (12.60, 19.20)	18.60 (15.00, 23.50)	22.40 (17.70, 29.00)	28.10 (21.20, 38.20)	< 0.001
AST (U/L)^b^	24.40 (21.00, 28.90)	22.50 (19.60, 26.10)	23.70 (20.70, 27.50)	25.00 (21.60, 29.20)	27.00 (22.90, 32.70)	< 0.001
FLI^a^	48.29 (30.07)	10.76 (4.98)	32.52 (7.66)	61.12 (8.53)	88.26 (6.97)	< 0.001
FLI ≥ 60	153,203 (38.6)	0 (0.0)	0 (0.0)	53,739 (53.9)	99,464 (100.0)	< 0.001
HSI^a^	35.60 (5.87)	30.21 (2.78)	33.42 (3.10)	36.48 (3.54)	42.25 (5.42)	< 0.001
HSI > 36	163,136 (41.3)	2012 (2.1)	19,663 (19.9)	52,180 (52.5)	89,281 (90.3)	< 0.001

Note: Numbers are *n* (%) unless otherwise stated

*IPAQ* International Physical Activity Questionnaire, *BMI* body mass index, *WC* waist circumstance, *TG* triglycerides, *GGT* gamma-glutamyltransferase, *ALT* alanine aminotransferase, *AST* aspartate aminotransferase, *FLI* fatty liver index, *HSI* hepatic steatosis index

^a^displayed as mean ± standard deviation

^b^displayed as median (interquartile range)

### Baseline non-alcoholic fatty liver and risk of incident IBS

During a total of 4,776,162 person-years’ follow-up, 7129 cases of incident IBS were identified. Cumulative incidence rate of IBS was 1.49 (95%CI: 1.46–1.53) per 1000 person-years. The 12-year cumulative incidence of IBS was 1.8% (95%CI: 1.7–1.9%), 1.7% (1.6–1.8%), and 1.8% (1.7–1.9%) in quartile 2, 3 and 4 groups versus 1.9% (1.8–2.0%) in the lowest quartile group. Cox proportional hazard regression model with restricted cubic spline indicated baseline FLI was linearly associated with risk of IBS (*P* = 0.383, Additional file 2: Fig. S1). Fatty liver was associated with a 21% risk increase of IBS (HR_Q4 VS Q1_ = 1.21, 95%CI: 1.13–1.30, *P*_trend_ < 0.001) according to fully adjusted model 3 (Table 2). Meanwhile, compared with the lowest quartile group (Q1), both FLI Q2 (adjusted HR = 1.12, 1.05–1.20) and Q3 (adjusted HR = 1.17, 1.10–1.26) groups were associated with a significantly higher risk of IBS.

**Table 2:** Risk of IBS according to Quartiles of baseline fatty liver index

	**Quartile 1**	**Quartile 2**	**Quartile 3**	**Quartile 4**	***P* for trend**
No. of participants	98,371	99,289	99,714	99,464	
No. of incident IBS	1882	1797	1701	1749	
Follow-up, person-years	1,192,803	1,197,965	1,199,435	1,185,959	
Follow-up, years					
Mean ± SD	12.1 ± 1.8	12.1 ± 1.8	12.0 ± 1.9	11.9 ± 2.1	
Median (IQR)	12.4 (11.7–13.1)	12.4 (11.6–13.1)	12.4 (11.6–13.1)	12.3 (11.6–13.1)	
Hazard ratio for incident IBS (95%CI, *P* value)					
Adjusted model 1	Reference	1.15 (1.08–1.23)	1.24 (1.16–1.33)	1.36 (1.27–1.46)	< 0.001
Adjusted model 2	Reference	1.13 (1.06–1.21)	1.20 (1.12–1.28)	1.27 (1.19–1.37)	< 0.001
Adjusted model 3	Reference	1.12 (1.05–1.20)	1.17 (1.10–1.26)	1.21 (1.13–1.30)	< 0.001

Note: Adjusted model 1: Age and gender were adjusted; Adjusted model 2: Townsend deprivation index, education level, ethnicity, smoking status, and alcohol drinking were additionally adjusted; Adjusted model 3: IPAQ (International Physical Activity Questionnaire), and type 2 diabetes were additionally adjusted; P for trend was calculated by using median value (10.5, 32.2, 61.2, and 88.6) of each fatty liver index quartile

*IBS* irritable bowel syndrome, *SD* standard deviation, *IQR* inter-quartile range

**Fig. 2:**
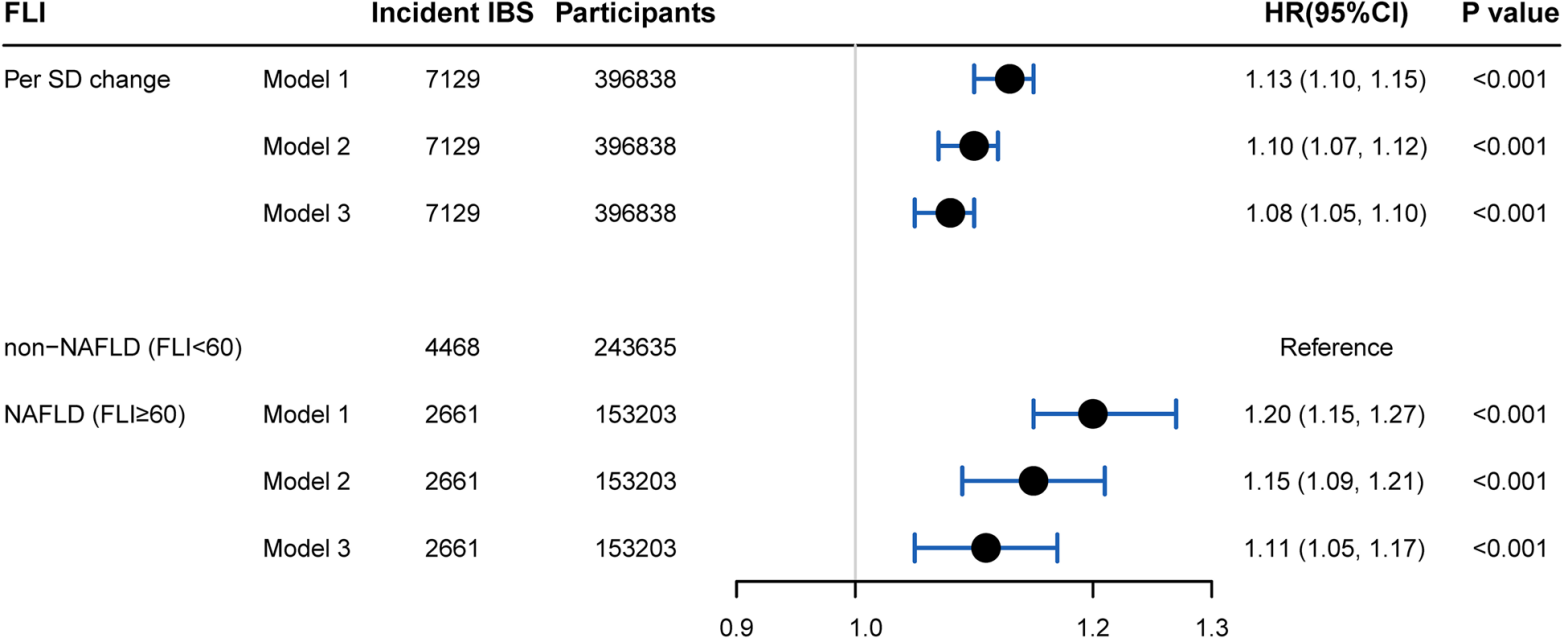
The association between baseline FLI and incident IBS.  Note: All adjusted HRs were calculated by adjusting the following covariates: age, gender, Townsend deprivation index, education level, ethnicity, smoking status, alcohol drinking, IPAQ (International Physical Activity Questionnaire), and type 2 diabetes. IBS: irritable bowel syndrome; FLI: fatty liver index; CI: confidence interval; HR: hazard ratio

Furthermore, totally, 2661(1.52 per 1000 person-years) and 4468 (1.45 per 1000 person-years) incident IBS occurred in NAFLD and non-NAFLD groups, respectively. Although 12-year cumulative incidence of IBS seemed similar between NAFLD (1.8%, 1.7–1.8%) and non-NAFLD (1.8%, 1.8–1.9%) via the Kaplan–Meier method, NAFLD patients showed a 13% higher risk of developing IBS (HR = 1.13, 1.05–1.17) after multivariable adjustment compared with non-NAFLD (Fig. 2). Moreover, either lean, non-lean, non-obese, or obese NAFLD patients had an increased risk of incident IBS, with a HR of 1.22 (95%CI: 0.92–1.60), 1.11 (1.05–1.17), 1.14 (1.06–1.22), and 1.09 (1.03–1.16), respectively (Additional file 2: Fig. S2). Additionally, an 8% increased risk was associated with per SD change of FLI (Fig. 2).

### Subgroup analysis

**Fig. 3:**
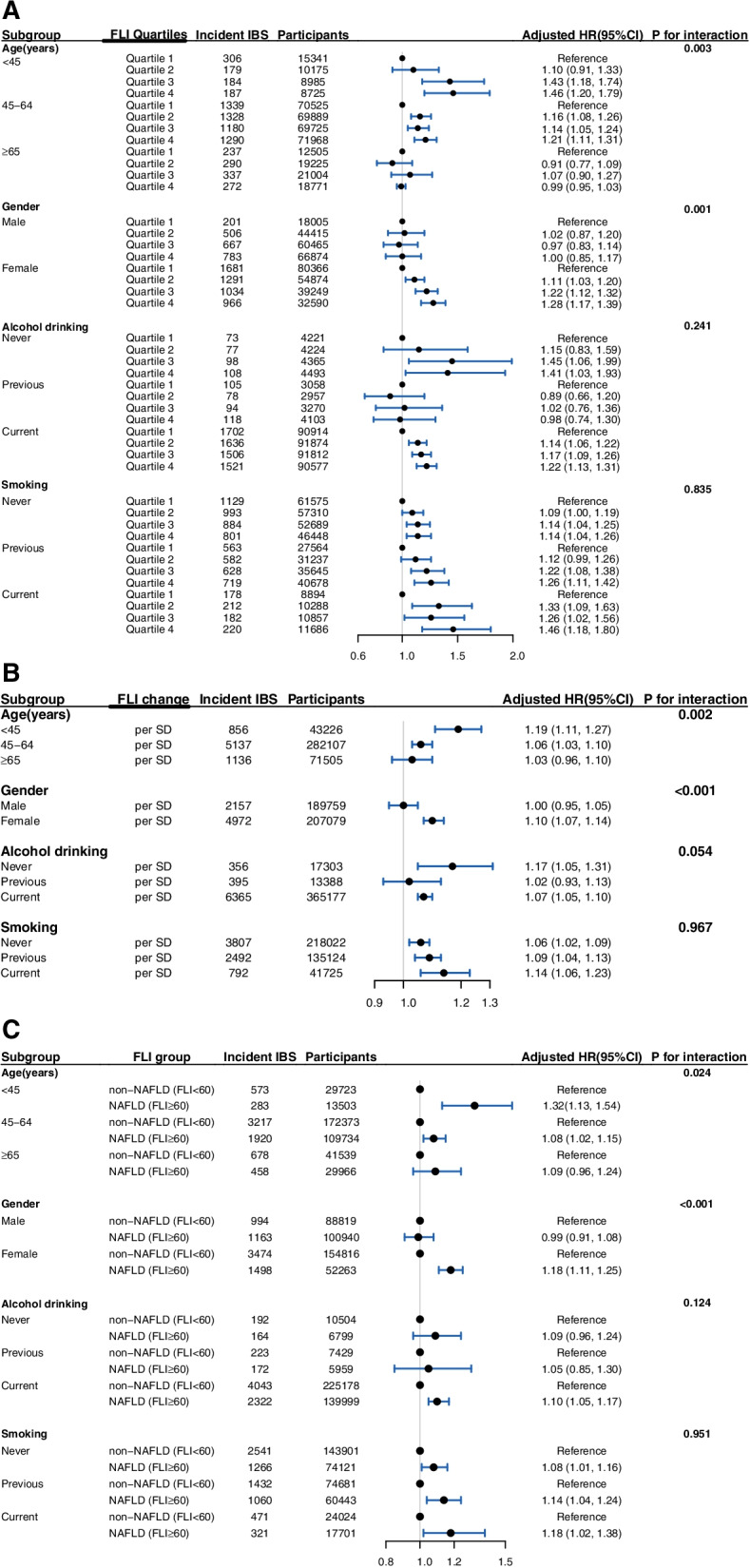
Subgroup analysis for the association between baseline FLI and incident IBS. **A** According to FLI quartiles. **B** According to FLI per SD change. **C** According to the diagnosis of NAFLD or not by the predefined cutoff of FLI. All adjusted HRs were calculated by adjusting the following covariates: age, gender, Townsend deprivation index, education level, ethnicity, smoking status, alcohol drinking, IPAQ (International Physical Activity Questionnaire) and type 2 diabetes; IBS: irritable bowel syndrome; FLI: fatty liver index; CI: confidence interval; HR: hazard ratio

In subgroup analysis, the increased IBS risk associated with FLI quartiles was generally observed across age, gender, alcohol drinking, and smoking subgroups, except for age ≥ 65 years old, male, and previous alcohol drinking subgroups (Fig. 3A). Moreover, we observed significant interactions across age/gender and FLI quartiles (*P* for interaction 0.003 for age and 0.001 for gender).

Similarly, consistent subgroup findings were observed when using per SD change and diagnosis of NAFLD or not by a predefined cutoff of FLI (Fig. 3B, C). Significant modification effects by age and gender were both detected when using per SD change (*P* for interaction 0.002 for age and < 0.001 for gender) and diagnosis of NAFLD or not (*P* for interaction 0.024 for age and < 0.001 for gender). The increased IBS risk was observed in females but not in males.

### Sensitivity analysis

Results of sensitivity analysis by quartiles, per SD change and diagnosis of NAFLD or not according to a predefined cutoff of FLI were similar to the main analysis, when excluding incident IBS cases within 1 year or 2 years after baseline, excluding incident ALD cases, excluding participants with hepatitis B/C virus seropositivity, performing competing risk model, additional adjusting depression and anxiety, or with the age-matched cohort as a dataset (Table 3, Additional file 1: Table S3). Moreover, results of sensitivity analysis by HSI, either treated as per SD change or diagnosis of NAFLD or not, were all consistent with principal findings (Additional file 1: Table S4).

**Table 3:** Sensitivity analysis regarding the risk of IBS according to Quartiles of baseline fatty liver index

FLI Quartiles	No. of IBS	No. of participants	Adjusted HR (95%CI)	*P* value	*P* for trend
Sensitivity analysis 1: excluding IBS participants diagnosed within 1 year after baseline (*N* = 396,184)					
Quartile 1	1691	98,180	Reference		< 0.001
Quartile 2	1659	99,151	1.15 (1.08–1.24)	< 0.001	
Quartile 3	1552	99,565	1.19 (1.11–1.28)	< 0.001	
Quartile 4	1573	99,288	1.21 (1.12–1.31)	< 0.001	
Sensitivity analysis 2: excluding IBS participants diagnosed within 2 years after baseline (*N* = 395,546)					
Quartile 1	1509	97,998	Reference		
Quartile 2	1495	98,987	1.16 (1.08–1.25)	< 0.001	< 0.001
Quartile 3	1405	99,418	1.20 (1.11–1.30)	< 0.001	
Quartile 4	1428	99,143	1.22 (1.13–1.32)	< 0.001	
Sensitivity analysis 3: excluding incident alcoholic liver disease participants after baseline (*N* = 395,775)					
Quartile 1	1878	98,306	Reference		
Quartile 2	1793	99,161	1.12 (1.05–1.20)	0.001	< 0.001
Quartile 3	1696	99,496	1.18 (1.10–1.26)	< 0.001	
Quartile 4	1737	98,812	1.21 (1.13–1.30)	< 0.001	
Sensitivity analysis 4: competing risk model (*N* = 396,838, No. of competing events = 24,742)					
Quartile 1	1882	98,371	Reference		
Quartile 2	1797	99,289	1.13 (1.05–1.20)	0.001	< 0.001
Quartile 3	1701	99,714	1.18 (1.10–1.26)	< 0.001	
Quartile 4	1749	99,464	1.21 (1.12–1.30)	< 0.001	
Sensitivity analysis 5: excluding HBV or HCV antigen-positive participants after baseline (*N* = 396,613)					
Quartile 1	1877	98,310	Reference		
Quartile 2	1796	99,235	1.13 (1.05, 1.20)	< 0.001	< 0.001
Quartile 3	1701	99,665	1.18 (1.10, 1.26)	< 0.001	
Quartile 4	1749	99,403	1.21 (1.13, 1.30)	< 0.001	
Sensitivity analysis 6: additionally adjusted psychologic disorder including depression and anxiety (*N* = 396,838)					
Quartile 1	1882	98,371	Reference		
Quartile 2	1797	99,289	1.11 (1.03–1.18)	0.003	< 0.001
Quartile 3	1701	99,714	1.14 (1.07–1.23)	< 0.001	
Quartile 4	1749	99,464	1.16 (1.08–1.24)	< 0.001	

Note: All adjusted HRs were calculated by adjusting the following covariates: age, gender, Townsend deprivation index, education level, ethnicity, smoking status, alcohol drinking, IPAQ (International Physical Activity Questionnaire) and type 2 diabetes. *P* for trend was calculated by using the median value of each fatty liver index Quartile (10.5, 32.2, 61.2, and 88.6 for sensitivity analysis 1, 2, 4, 5, and 6; 10.5, 32.2, 61.2, and 88.5 for sensitivity analysis 3)

*IBS* irritable bowel syndrome, *HR* hazard ratio, *CI* confidence interval

## Discussion

In this prospective cohort study with a long-term follow-up of 0.4 million adults, participants with the highest quartile of the fatty liver index had a 21% increased risk of IBS occurrence. Participants with NAFLD diagnosis had a 13% higher risk of developing IBS. The positive association was particularly observed in females, but not in males.

Given the rising incidence of NAFLD globally during recent decades, our results may partially explain the current upward trend of IBS [3, 7, 8]. An epidemiological projection indicates there would be an expected increase of nearly 120 million people living with IBS between 2020 and 2040 worldwide [26]. Non-alcoholic fatty liver degree as well as NAFLD might be etiologically associated with IBS occurrence. If confirmed by future studies, the findings may have profound public health significance for the prevention of IBS. An estimated 7.3% (95% CI, 4.2–10.3%) of all IBS cases would be avoided if all UKB cohort members decreased their baseline FLI by more than 27. Particularly, approximately 10.3% (95% CI, 6.3–14.3%) of all IBS cases among women would have been avoided if baseline FLI decreased by more than 27 among study participants. Hence, if applied to the current general population, a considerable amount of health resources and medical cost related to IBS would have been saved.

The biological mechanisms for the positive association of a high degree of non-alcoholic fatty liver and NAFLD with incident IBS remain to be fully elucidated. Since NAFLD pathogenesis has been mainly considered as liver fat accumulation and subsequent hepatic inflammation based on “two-hit” theory, hepatic fat accumulation and inflammation along with immune system activation are hypothetically implicated in IBS development [16, 17, 27]. Several studies discovered similar trends of shared proinflammatory cytokines in both IBS and NAFLD, including increased expressions of TNF-α, IL-6, IL-8, and IL-1β, as well as decreased levels of IL-10 in vitro, animal models, and human studies [28–32]. Although the underlying mechanism was unclear, those cytokines have been reported to play important roles via Toll-like receptors (TLR) in the development of IBS symptoms, including the effect on peripheral and central nervous systems to develop hypersensitivity and gut hypomotility (TNF-α), stimulation of gut submucosal neurons (IL-6), intestinal barrier integrity (TNF-α, IL-6) and maintenance of gut homeostasis (IL-1β, IL-6, IL-10) [16, 17, 29].

Furthermore, the interaction between gut and liver primarily through the portal vein and biliary tract has attracted increasing attention recently, the so-called gut-liver axis [33, 34]. It has been reported bile salts and antimicrobial molecules are transported from the liver to the intestinal lumen via the biliary tract to maintain gut eubiosis by controlling unrestricted bacterial overgrowth [13, 33]. As diseased fatty liver could not effectively inhibit the overgrowth of bacteria, harmful microbial by-products could not be removed timely, which may further aggravate gut dysbiosis. Increasing evidence has revealed the involvement of intestinal dysbiosis in IBS pathogenesis [35, 36]. Alternation of gut microbiota (i.e., increased Clostridium and decreased lactobacilli with bifidobacterial) was associated with impaired intestinal permeability, impaired intestinal motility, and visceral hypersensitivity, which may contribute to the development of IBS symptoms [33–36].

Additionally, communication between gut and liver has been reported further transmitted from liver to brain via the autonomic nervous system, indicating an involvement of liver-brain-gut neural arc [13–15]. Recent experimental evidence demonstrated novel vago-vagal liver-brain-gut reflex arc mediated the differentiation of colonic peripheral regulatory T cells (pT_reg_ cells), which could maintain immune homeostasis and thereby prevent excessive inflammatory response [14]. A mouse study revealed hepatic vagal sensory afferent nerves could reduce colonic pT_reg_ cell pool through activation of muscarinic acetylcholine receptors once left vagal sensory afferents from liver to brainstem was disrupted, leading to disturbance of intestinal barrier and further susceptibility of IBS [14]. Despite recent advances in understanding of liver-brain-gut interaction, more investigation is needed to further clarify related potential mechanisms.

Interestingly, a positive association between non-alcoholic fatty liver and risk of IBS was observed in females rather than males in our study. Despite IBS is developed predominantly in females, the sex-gender difference in incident IBS still remains largely unknown. Recent data suggested interaction of trace aminergic signaling and sex hormones, especially female reproductive hormones, may play a critical role in IBS genesis [37, 38]. Disturbance of trace aminergic system might result in altered colonic ion secretion, hyperreactivity of the immune system, and fluctuations of 5-hydroxytryptamine (5-HT) levels, thereby leading to disruption of the gut microbiome, and mucosal immunity, all of which are implicated as etiological factors in IBS pathogenesis [37, 38]. Further studies are needed to confirm our findings and elucidate possible mechanisms.

To the best of our knowledge, there are no studies highlighting the link between non-alcoholic fatty liver degree as well as NAFLD and incident IBS. Based on the well-designed, large-scaled population-based cohort with the longest follow-up to date, non-alcoholic fatty liver degree measured in different approaches (i.e., NAFLD or not, quartiles, per SD change) was examined in detail and all were consistently observed to associate with increased risk of IBS. Moreover, multiple important lifestyle confounders, including age, gender, alcohol, smoking, physical activity, and socioeconomic status, were thoroughly adjusted. Additionally, various sensitivity analyses by accounting for protopathic bias and misclassification bias, and further substantial subgroup analyses were conducted, verifying the robustness of the results.

Several limitations should be considered. Firstly, NAFLD was measured by estimated indices in this study, rather than the gold diagnostic criteria including hepatic image or histology from liver biopsy due to unavailable data, which might lead to potential measurement error. However, FLI has shown excellent performance with transient elastography determined NAFLD and has been widely accepted as a reasonable substitute for obtaining population estimates [20, 21]. Meanwhile, our sensitivity analysis considering HSI as a measurement of NAFLD [24] indicated the robustness of principal findings. In addition, risk estimates would be attenuated and toward to the null association even if this non-differential measurement error existed, which instead supported our positive associations. Secondly, residual confounders cannot be ruled out since some potential covariates, either unmeasured or unknown, may confound the association between NAFLD and IBS, although we have carefully controlled for numerous potential confounders. Thirdly, IBS was identified according to ICD-10 codes in primary care or hospitalized medical records, rather than via structured questionnaire (i.e., Rome III or IV scale) or interview. Since some IBS cases in the community may not take medical consultation, a proportion of IBS cases in this large population-based cohort might remain undiagnosed, leading to an underestimation of the IBS incidence rate. However, underdiagnosis of IBS would exist in both exposure (i.e., NAFLD or quartile 2–4 groups) and non-exposure groups (i.e., non-NAFLD or quartile 1 group). Thus, the association would be underestimated under the circumstances of probably non-differential misclassification bias, which would in turn support our positive findings. Fourthly, non-alcoholic fatty liver was only assessed at baseline. Thus, a change of fatty liver degree during the follow-up could not be obtained. Accordingly, the association between changes in NAFLD and the risk of IBS could not be evaluated. Finally, we were not able to further examine the association between NAFLD and the development of different IBS subtypes due to the unavailability of such data in UKB. Future cohort studies are needed to elucidate associated risk with IBS subtypes.

## Conclusions

In summary, in this large-scale prospective cohort study of the UK population, a high degree of non-alcoholic fatty liver as well as non-alcoholic fatty liver disease was associated with an increased risk of incident IBS. However, these findings are needed to be confirmed by further well-designed prospective cohort studies and trials in the diverse ethnic population. Future animal and experimental research are also warranted to elucidate the underlying biological mechanisms.

## Supplementary Information

**Additional file 1. Table S1.** ICD codes and Field ID definingdiseases in UKB. **Table S2.** Baseline characteristicsaccording to diagnosis of NAFLD or not by predefined cutoff of FLI in UKBiobank cohort. **Table S3.**Sensitivity analysis regarding risk of IBS according to per SD change anddiagnosis of NAFLD or not by predefined cutoff of FLI. **Table S4.** Sensitivityanalysis regarding risk of IBS according to per SD change and diagnosis ofNAFLD or not by predefined cutoff of baseline hepatic steatosis index.

**Additional file 2. FigureS1.** Restricted cubic splinefor the association of baseline fatty liver index with incident IBS. **Figure S2.** Risk of incident IBSassociated with NAFLD type (lean, non-obese and obese NAFLD).

## Data Availability

All data relevant to the study were using the UK Biobank Resource under application number [74444]. No additional data available.

## Abbreviations

5-HT: 5-Hydroxytryptamine
ALD: Alcoholic liver disease
ALT: Serum alanine aminotransferase
AST: Aspartate aminotransferase
AUROC: Area under the receiver operator curve
CI: Confidence interval
FLI: Fatty liver index
GGT: Gamma-glutamyltransferase
HSI: Hepatic steatosis index
IBD: Inflammatory bowel disease
IBS: Irritable bowel syndrome
ICD-10: International Classification of Disease-10
IL: Interleukin
IPAQ: International Physical Activity Questionnaire
NAFLD: Non-alcoholic fatty liver disease
SD: Standard deviation
TG: Triglycerides
TLR: Toll-like receptors
TNF: Tumor necrosis factor
UKB: UK Biobank
WC: Waist circumstance
