# Supplementary material for: Non-alcoholic fatty liver is associated with increased risk of irritable bowel syndrome: a prospective cohort study
Source: BMC Med. 2022 Aug 22;20:262. doi: 10.1186/s12916-022-02460-8 (PMC9394037; doi:10.1186/s12916-022-02460-8)
Supplement: Supplementary file 1 — Additional file 1. Table S1. ICD codes and Field ID definingdiseases in UKB. Table S2. Baseline characteristicsaccording to diagnosis of NAFLD or not by predefined cutoff of FLI in UKBiobank cohort. Table S3.Sensitivity analysis regarding risk of IBS according to per SD change anddiagnosis of NAFLD or not by predefined cutoff of FLI. Table S4. Sensitivityanalysis regarding risk of IBS according to per SD change and diagnosis ofNAFLD or not by predefined cutoff of baseline hepatic steatosis index. [file 12916_2022_2460_MOESM1_ESM.docx]

**Non-alcoholic fatty liver is associated with increased risk of irritable bowel syndrome: a prospective cohort study**

**Additional file 1**

**Table S1.** **ICD codes and Field ID defining diseases in UKB.**

| **Disease** | **ICD-10** | **ICD-9** | **Data field ID in UKB** |
| --- | --- | --- | --- |
| Irritable bowel syndrome | K58 | - | 131638 |
| Alcoholic liver disease | K70 | 5710 | 131658 |
| Ulcerative colitis | K51 | - | 131628 |
| Crohn’s disease | K50 | - | 131626 |
| Coeliac disease | K90.0 | 5790 | 41270, 41202, 41271, 41203 |

Note: ICD: international classification disease.

**Table S2. Baseline characteristics according to diagnosis of NAFLD or not by predefined cutoff of FLI in UK Biobank cohort.**

| **Characteristic** | **Total**  **(N=396838)** | **Non-NAFLD**  **(N=243635)** | **NAFLD**  **(N=153203)** | **P value** |
| --- | --- | --- | --- | --- |
| Age(years)^*^ | 56.22±8.11 | 55.76±8.23 | 56.97±7.87 | <0.001 |
| Gender |  |  |  | <0.001 |
| Male | 189759 (47.8) | 88819 (36.5) | 100940 (65.9) |  |
| Female | 207079 (52.2) | 154816 (63.5) | 52263 (34.1) |  |
| Ethnicity |  |  |  | 0.017 |
| Non-White | 22788 (5.7) | 13953 (5.7) | 8835 (5.8) |  |
| White | 372582 (93.9) | 228833 (93.9) | 143749 (93.8) |  |
| Unknown | 1468 (0.4) | 849 (0.3) | 619 (0.4) |  |
| Education level |  |  |  | <0.001 |
| Non-university | 261356 (65.9) | 151621 (62.2) | 109735 (71.6) |  |
| University | 130800 (33.0) | 89470 (36.7) | 41330 (27.0) |  |
| Unknown | 4682 (1.2) | 2544 (1.0) | 2138 (1.4) |  |
| Townsend deprivation index | | | | |
| Mean (SD) | -1.30 (3.09) | -1.47 (3.00) | -1.04 (3.20) | <0.001 |
| Q1(≤ -3.63) | 99950 (25.2) | 64686 (26.6) | 35264 (23.0) | <0.001 |
| Q2(-3.63 - -2.12) | 99303 (25.0) | 62622 (25.7) | 36681 (23.9) |  |
| Q3(-2.12 - 0.58) | 99259 (25.0) | 60722 (24.9) | 38537 (25.2) |  |
| Q4 (>0.58) | 97832 (24.7) | 55305 (22.7) | 42527 (27.8) |  |
| Unknown | 494 (0.1) | 300 (0.1) | 194 (0.1) |  |
| Smoking status |  |  |  | <0.001 |
| Never | 218022 (54.9) | 143901 (59.1) | 74121 (48.4) |  |
| Previous | 135124 (34.1) | 74681 (30.7) | 60443 (39.5) |  |
| Current | 41725 (10.5) | 24024 (9.9) | 17701 (11.6) |  |
| Unknown | 1967 (0.5) | 1029 (0.4) | 938 (0.6) |  |
| Alcohol drinking |  |  |  | <0.001 |
| Never | 17303 (4.4) | 10504 (4.3) | 6799 (4.4) |  |
| Previous | 13388 (3.4) | 7429 (3.0) | 5959 (3.9) |  |
| Current | 365177 (92.0) | 225178 (92.4) | 139999 (91.4) |  |
| Unknown | 970 (0.2) | 524 (0.2) | 446 (0.3) |  |
| IPAQ |  |  |  | <0.001 |
| Low | 59637 (15.0) | 30172 (12.4) | 29465 (19.2) |  |
| Moderate | 131111 (33.0) | 81280 (33.4) | 49831 (32.5) |  |
| High | 131356 (33.1) | 88150 (36.2) | 43206 (28.2) |  |
| Unknown | 74734 (18.8) | 44033 (18.1) | 30701 (20.0) |  |
| BMI |  |  |  | <0.001 |
| <18.5 kg/m^2^ | 1896 (0.5) | 1894 (0.8) | 2 (0.0) |  |
| 18.5-24.9 kg/m^2^ | 124057 (31.3) | 120878 (49.6) | 3179 (2.1) |  |
| 25.0-29.9 kg/m^2^ | 171674 (43.3) | 110039 (45.2) | 61635 (40.2) |  |
| ≥30 kg/m^2^ | 99211 (25.0) | 10824 (4.4) | 88387 (57.7) |  |
| Diabetes | 10014 (2.5) | 2455 (1.0) | 7559 (4.9) | <0.001 |
| WC (cm) ^*^ | 90.47 (13.43) | 82.82 (8.93) | 102.63 (9.98) | <0.001 |
| TG (mg/dL) ^*^ | 154.40(91.02) | 118.19 (54.50) | 211.99 (106.46) | <0.001 |
| GGT (U/L) ^#^ | 26.40  (18.60, 41.00) | 21.20  (16.20, 29.20) | 39.70  (28.00, 61.00) | <0.001 |
| ALT (U/L) ^#^ | 20.25  (15.47, 27.57) | 17.60  (14.10, 22.60) | 26.30  (20.00, 35.50) | <0.001 |
| AST (U/L) ^#^ | 24.40  (21.00, 28.90) | 23.40  (20.30, 27.20) | 26.40  (22.50, 31.60) | <0.001 |
| FLI^*^ | 48.29 (30.07) | 27.63 (16.86) | 81.14 (11.52) | <0.001 |
| HSI^*^ | 35.60 (5.87) | 32.53 (3.65) | 40.50 (5.39) | <0.001 |
| FLI Quartile |  |  |  | <0.001 |
| Quartile 1 | 98371 (24.8) | 98371 (40.4) | 0 (0.0) |  |
| Quartile 2 | 99289 (25.0) | 99289 (40.8) | 0 (0.0) |  |
| Quartile 3 | 99714 (25.1) | 45975 (18.9) | 53739 (35.1) |  |
| Quartile 4 | 99464 (25.1) | 0 (0.0) | 99464 (64.9) |  |

Note: Numbers are n (%) unless otherwise stated. *: displayed as mean±standard deviation. #: displayed as median (interquartile range). IPAQ: International Physical Activity Questionnaire; BMI: body mass index. WC: waist circumstance. TG: triglycerides. GGT: Gamma glutamyltransferase. ALT: alanine aminotransferase. AST: aspartate aminotransferase. FAI: fatty liver index. HSI: hepatic steatosis index. NAFLD was defined as FLI≥60, and non-NAFLD was defined as FLI<60. NAFLD: non-alcoholic liver disease.

**Table S3.** **Sensitivity analysis regarding risk of IBS according to per SD change and diagnosis of NAFLD or not by predefined cutoff of FLI.**

| **FLI** | **No. of IBS** | **No. of participants** | **adjusted HR (95%CI)** | **P value** |
| --- | --- | --- | --- | --- |
| Sensitivity analysis 1: excluding IBS participants diagnosed within 1 year after baseline (N=396184) | | | | |
| Per SD change | 6475 | 396184 | 1.07 (1.05, 1.10) | <0.001 |
| Non-NAFLD (FLI<60) | 4061 | 243228 | Reference |  |
| NAFLD (FLI≥60) | 2414 | 152956 | 1.11 (1.05, 1.17) | <0.001 |
| Sensitivity analysis 2: excluding IBS participants diagnosed within 2 years after baseline (N=395546) | | | | |
| Per SD change | 5837 | 399546 | 1.08 (1.05, 1.11) | <0.001 |
| Non-NAFLD (FLI<60) | 3645 | 242812 | Reference |  |
| NAFLD (FLI≥60) | 2192 | 152734 | 1.11 (1.05, 1.18) | <0.001 |
| Sensitivity analysis 3: excluding incident alcoholic liver disease participants after baseline (N=395775) | | | | |
| Per SD change | 7104 | 395775 | 1.08 (1.05, 1.10) | <0.001 |
| Non-NAFLD (FLI<60) | 4460 | 243362 | Reference |  |
| NAFLD (FLI≥60) | 2644 | 152413 | 1.11 (1.05, 1.17) | <0.001 |
| Sensitivity analysis 4: competing risk model (N=396838, No. of competing events=24742) | | | | |
| Per SD change | 7129 | 396838 | 1.07 (1.05, 1.10) | <0.001 |
| Non-NAFLD (FLI<60) | 4468 | 243635 | Reference |  |
| NAFLD (FLI≥60) | 2661 | 153203 | 1.11 (1.05, 1.17) | <0.001 |
| Sensitivity analysis 5: excluding HBV or HCV antigen positive participants (N=396613) | | | | |
| Per SD change | 7123 | 396613 | 1.08 (1.05, 1.10) | <0.001 |
| Non-NAFLD (FLI<60) | 4462 | 243496 | Reference |  |
| NAFLD (FLI≥60) | 2661 | 153117 | 1.11 (1.05, 1.17) | <0.001 |
| Sensitivity analysis 6: additionally adjusted psychologic disorder including depression and anxiety (N=396838) | | | | |
| Per SD change | 7129 | 396838 | 1.06 (1.03, 1.08) | <0.001 |
| Non-NAFLD (FLI<60) | 4468 | 243635 | Reference |  |
| NAFLD (FLI≥60) | 2661 | 153203 | 1.08 (1.02, 1.14) | 0.004 |
| Sensitivity analysis 7: age-matched cohort between NAFLD and non-NAFLD (1:1 matching, N=306406) | | | | |
| Non-NAFLD (FLI<60) | 2833 | 153203 | Reference |  |
| NAFLD (FLI≥60) | 2661 | 153203 | 1.07 (1.01, 1.13) | 0.024 |

Note: All adjusted HRs were calculated by adjusting the following covariates: age, gender, Townsend deprivation index, education level, ethnicity, smoking status, alcohol drinking, IPAQ (International Physical Activity Questionnaire) and type 2 diabetes. IBS: irritable bowel syndrome; HR: hazard ratio. CI: confidence interval. FLI: fatty liver index. NAFLD: non-alcoholic liver disease.

**Table S4.** **Sensitivity analysis regarding risk of IBS according to per SD change and diagnosis of NAFLD or not by predefined cutoff of baseline hepatic steatosis index.**

| **HSI** | **No. of IBS** | **No. of participants** | **adjusted HR (95%CI)** | **P value** |
| --- | --- | --- | --- | --- |
| Sensitivity analysis 1: excluding participants with missing baseline HSI (N=395836) | | | | |
| Per SD change | 7107 | 395836 | 1.03 (1.01, 1.05) | 0.020 |
| Non-NAFLD (HSI≤36) | 3967 | 232436 | Reference |  |
| NAFLD (HSI>36) | 3140 | 163400 | 1.07 (1.02, 1.12) | 0.005 |
| Sensitivity analysis 2: excluding IBS participants diagnosed within 1 year after baseline in dataset for sensitivity analysis 1 (N=395185) | | | | |
| Per SD change | 6456 | 395185 | 1.03 (1.01, 1.05) | 0.022 |
| Non-NAFLD (HSI≤36) | 3606 | 232075 | Reference |  |
| NAFLD (HSI>36) | 2850 | 163110 | 1.07 (1.02, 1.12) | 0.010 |
| Sensitivity analysis 3: excluding IBS participants diagnosed within 2 years after baseline in dataset for sensitivity analysis 1 (N=394550) | | | | |
| Per SD change | 5821 | 394550 | 1.03 (1.01, 1.06) | 0.018 |
| Non-NAFLD (HSI≤36) | 3247 | 231716 | Reference |  |
| NAFLD (HSI >36) | 2574 | 162834 | 1.07 (1.01, 1.13) | 0.017 |
| Sensitivity analysis 4: excluding incident alcoholic liver disease participants after baseline in dataset for sensitivity analysis 1 (N=394755) | | | | |
| Per SD change | 7082 | 394755 | 1.03 (1.01, 1.05) | 0.018 |
| Non-NAFLD (HSI≤36) | 3955 | 231894 | Reference |  |
| NAFLD (HSI >36) | 3127 | 162861 | 1.07 (1.02, 1.12) | 0.005 |
| Sensitivity analysis 5: competing risk model in dataset for sensitivity analysis 1 (N=395836, No. of competing events=24742) | | | | |
| Per SD change | 7107 | 395836 | 1.03 (1.01, 1.05) | 0.018 |
| Non-NAFLD (HSI≤36) | 3967 | 232436 | Reference |  |
| NAFLD (HSI >36) | 3140 | 163400 | 1.07 (1.02, 1.13) | 0.004 |
| Sensitivity analysis 6: excluding HBV or HCV antigen positive participants in dataset for sensitivity analysis 1 (N=395612) | | | | |
| Per SD change | 7101 | 395612 | 1.03 (1.01, 1.05) | 0.017 |
| Non-NAFLD (HSI≤36) | 3961 | 232310 | Reference |  |
| NAFLD (HSI >36) | 3140 | 163302 | 1.07 (1.02, 1.13) | 0.004 |

Note: All adjusted HRs were calculated by adjusting the following covariates: age, gender, Townsend deprivation index, education level, ethnicity, smoking status, alcohol drinking, IPAQ (International Physical Activity Questionnaire) and type 2 diabetes. IBS: irritable bowel syndrome. HR: hazard ratio. CI: confidence interval. HSI: hepatic steatosis index. NAFLD: non-alcoholic liver disease. HBV: hepatitis B virus. HCV: hepatitis C virus.
