# Supplementary material for: Non-alcoholic fatty liver is associated with increased risk of irritable bowel syndrome: a prospective cohort study
Source: BMC Med. 2022 Aug 22;20:262. doi: 10.1186/s12916-022-02460-8 (PMC9394037; doi:10.1186/s12916-022-02460-8)
Supplement: Supplementary file 2 — Additional file 2. FigureS1. Restricted cubic splinefor the association of baseline fatty liver index with incident IBS. Figure S2. Risk of incident IBSassociated with NAFLD type (lean, non-obese and obese NAFLD). [file 12916_2022_2460_MOESM2_ESM.docx]

**Non-alcoholic fatty liver is associated with increased risk of irritable bowel syndrome: a prospective cohort study**

**Additional file 2**

**Figure S1. Restricted cubic spline for the association of baseline fatty liver index with incident IBS.**

**
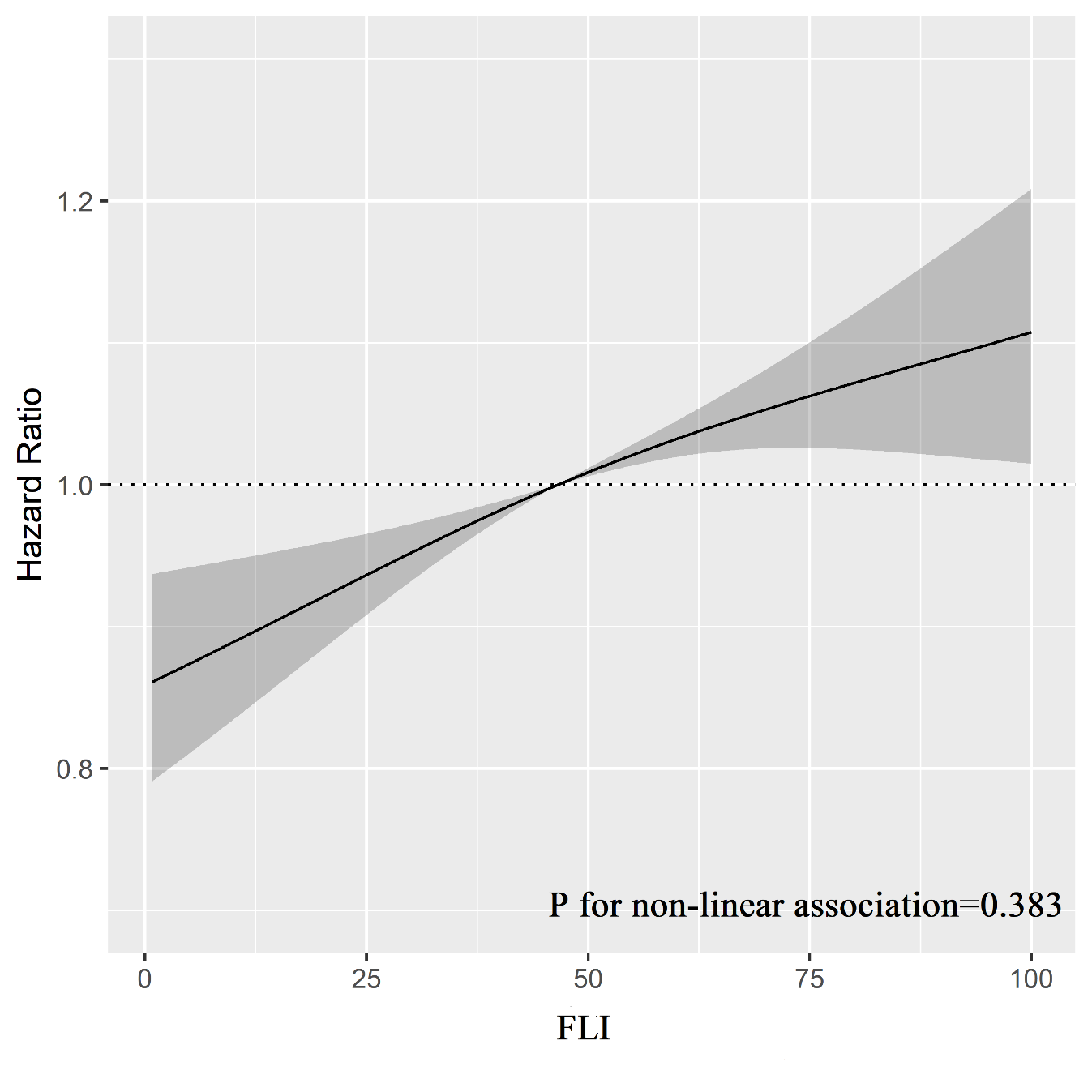
**

Note: reference point is the median value of baseline fatty liver index (46.55), after adjusting age, gender, Townsend deprivation index, education level, ethnicity, smoking status, alcohol drinking, IPAQ (International Physical Activity Questionnaire) and type 2 diabetes. Hazard ratios are indicated by solid line and 95% confidence intervals by shadow

area, with knots placed at 10th, 50th, and 90th percentiles. IBS: irritable bowel syndrome; HR: hazard ratio. CI: confidence interval. FLI: fatty liver index.

**Figure S2. Risk of incident IBS associated with NAFLD type (lean, non-obese and obese NAFLD).**

**
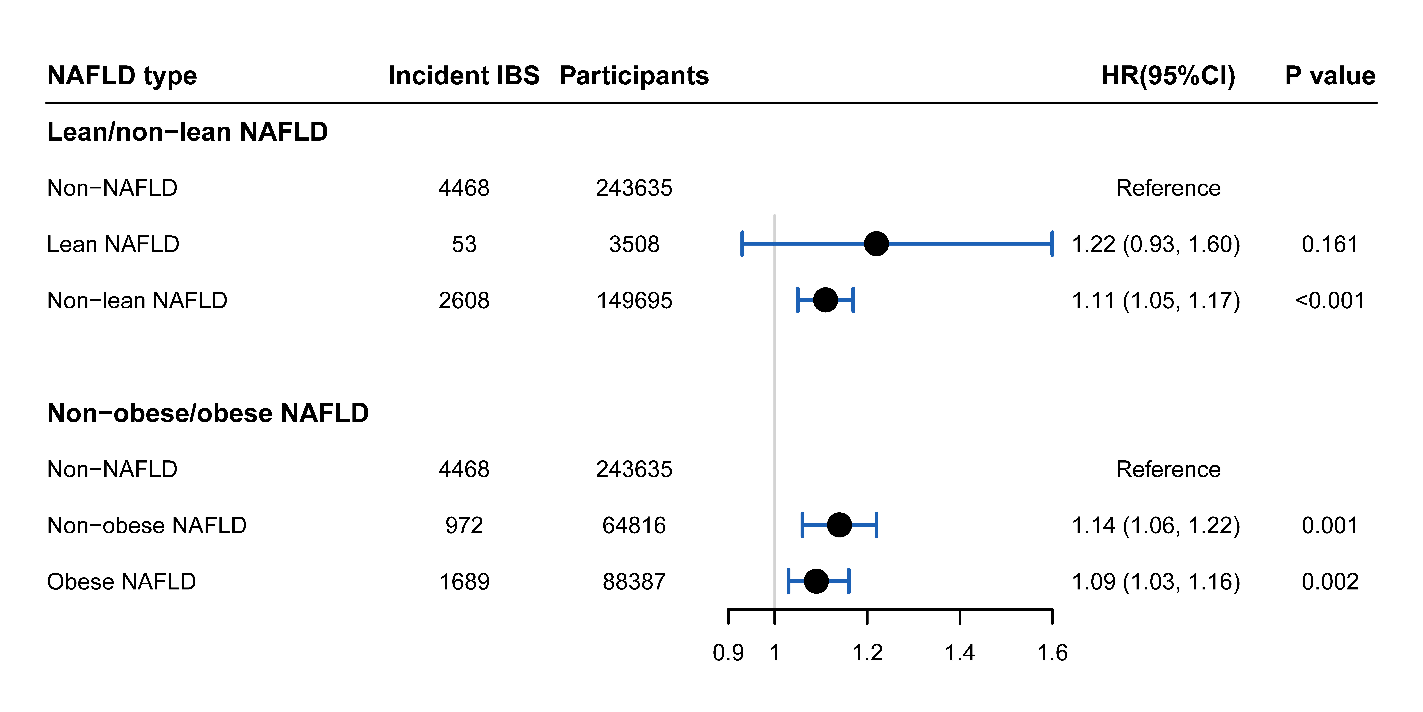
**

Note: Participants without NAFLD were considered as the reference group. All adjusted HRs were calculated by adjusting the following covariates: age, gender, Townsend deprivation index, education level, ethnicity, smoking status, alcohol drinking, IPAQ (International Physical Activity Questionnaire) and type 2 diabetes. IBS: irritable bowel syndrome; HR: hazard ratio; CI: confidence interval.
